# Transcriptional Regulation of Aflatoxin Biosynthesis and Conidiation in *Aspergillus flavus* by *Wickerhamomyces anomalus* WRL-076 for Reduction of Aflatoxin Contamination

**DOI:** 10.3390/toxins11020081

**Published:** 2019-02-01

**Authors:** Sui Sheng T. Hua, Siov Bouy L. Sarreal, Perng-Kuang Chang, Jiujiang Yu

**Affiliations:** 1 U.S. Department of Agriculture, Agricultural Research Service, Western Regional Research Center, 800 Buchanan Street, Albany, CA 94710, USA; Siov.Sarreal@ars.usda.gov; 2 U.S. Department of Agriculture, Agricultural Research Service, Southern Regional Research Center, 1100 Robert E. Boulevard, New Orleans, LA 70124, USA; PerngKuang.Chang@ars.usda.gov; 3 U.S. Department of Agriculture, Agricultural Research Service, Beltsville Agricultural Center, Beltsville, MD 70124, USA; jiujiang.yu8@gmail.com

**Keywords:** biocontrol mechanism, gene regulation, aflatoxin, conidiation, food safety, *Wickerhamomyces anomalus*, *Aspergillus flavus*

## Abstract

*Aspergillus flavus* is a ubiquitous saprophytic fungus found in soils across the world. The fungus is the major producer of aflatoxin (AF) B_1_, which is toxic and a potent carcinogen to humans. Aflatoxin B_1_ (AFB_1_) is often detected in agricultural crops such as corn, peanut, almond, and pistachio. It is a serious and recurrent problem and causes substantial economic losses. *Wickerhamomyces anomalus* WRL-076 was identified as an effective biocontrol yeast against *A. flavus*. In this study, the associated molecular mechanisms of biocontrol were investigated. We found that the expression levels of eight genes, *aflR*, *aflJ*, *norA*, *omtA*, *omtB*, *pksA*, *vbs*, and *ver-1* in the aflatoxin biosynthetic pathway cluster were suppressed. The decreases ranged from several to 10,000 fold in fungal samples co-cultured with *W. anomalus*. Expression levels of conidiation regulatory genes *brlA*, *abaA*, and *wetA* as well as sclerotial regulatory gene (*sclR*) were all down regulated. Consistent with the decreased gene expression levels, aflatoxin concentrations in cultural medium were reduced to barely detectable. Furthermore, fungal biomass and conidial number were significantly reduced by 60% and more than 95%, respectively. The results validate the biocontrol efficacy of *W. anomalus* WRL-076 observed in the field experiments.

## 1. Introduction

*Aspergillus flavus* is a saprophytic and pathogenic fungus. Many isolates of *A. flavus* produce the hepatocarcinogenic aflatoxin (AF) B_1_, which is often detected in agricultural crops including corn, cotton, peanuts, and tree nuts, and in many dried fruits and spices. AFB_1_ contamination in food results in substantial economic losses worldwide [1,2,3,4,5,6,7]. Therefore, more than 100 countries have established specific regulation guidelines limiting allowable amounts of AFB_1_ in foodstuffs [8,9]. Major importers of agricultural commodities have imposed threshold levels for AFB_1_ below 10 µg/kg, and these restrictions have had a major negative impact on the exportability of a number of crops [10].

The antifungal properties of *Pichia anomala* makes the species a suitable biocontrol agent against pathogenic and mycotoxigenic microorganisms in agricultural crops. Kurtzman [11] has recently renamed the species as *Wickerhamomyces anomalus*. Several *P. anomala* strains, namely strain J121 and strain K have been demonstrated to control storage mold in small grains [12,13,14] and to reduce fruit rot in apple [15,16]. In addition, *P. anomala* J121 has also been demonstrated to prevent ochratoxin accumulation by *Penicillium roqueforti* [17]. *Pichia anomala* strain WRL-076 was discovered as an effective antagonist against *A. flavus* through a visual bioassay [18]. The biocontrol efficacy of WRL-076 was evaluated further on pistachio flowers, leaves, nut-fruits, almond leaves, and corn. Spore production of *A. flavus* was reduced by about 80% in a pistachio orchard sprayed with the yeast [19,20,21,22]. Field experiments conducted in Texas [23] indicated that *P. anomala* significantly reduced the level of pre-harvest aflatoxin in corn by as much as 70%. The report also demonstrates a decreasing trend of aflatoxin levels with *P. anomala* treatments, and two applications of *P. anomala* at the silk stage of corn resulted in a significant reduction in aflatoxin accumulation (*p* < 0.05). One of the liquid formulations developed for *P. anomala* WRL-076 can preserve cell viability by up to 83%, even after cold storage for 12 months. In that formulation, the intracellular sorbitol and trehalose concentrations were high, and synergistically enhanced yeast viability up to 12 months [24].

However, the molecular mechanisms underlying the growth inhibition of *A. flavus* by strain *P*. *anomala* WRL-076 are not known. A previous study suggested that reduced metabolic function in conjunction with cell wall damage of *A. flavus* in a co-culture with *P. anomala* WRL-076 hinder *A. flavus* growth and biomass production [25]. *Pichia anomala* WRL-076 produces a major volatile compound, 2-phenylethanol, which inhibits the growth and expression of aflatoxin biosynthetic genes of *A. flavus* [26]. The suppression of aflatoxin biosynthesis in *A. flavus* by 2-phenylethanol appears to be associated with decreased activities in the degradation of branched-chain amino acids as revealed by a transcriptome study [27].

Isolates of *A. flavus* collected from agricultural fields are commonly categorized into two morphotypes based on the production of sclerotia, mycelial aggregates as an overwinter structure, and aflatoxin. One type is called S-strain that has sclerotia with diameters smaller than 400 μm. and another type is called L-strain that has sclerotia with diameters larger than 400 μm. S-strain isolates produce much more sclerotia and significantly less conidia than L-strain isolates cultured on the same solid media [28]. All S-strain isolates produce relatively higher amounts of aflatoxin B_1_ (AFB_1_) compared to L-strain isolates. The divergence between S- and L-strains probably occurred one to three million years ago [29]. A most recent comparative genomics study of *A. flavus* S- and L-morphotypes has yielded insights into their respective niche adaptation [30].

Genes directly involved in AF biosynthesis are clustered in an 80-kb genome region in *A. flavus* [31,32,33]. Regulation of the AF biosynthetic gene cluster is mediated by a complex network of global regulators and pathway-specific transcription factors [34,35,36,37,38]. *A. flavus* disseminates primarily via asexual spores (conidia), of which formation and maturation are governed by the central genetic regulatory circuit including BrlA, AbaA, and WetA [39,40,41,42,43,44]. SclR, a transcription factor, regulates hyphal morphology and sclerotial formation [45]. While conidia allow the fungus to mass-disseminate, sclerotia ensure survival in harsh environmental conditions in soil and germinate once conditions improve [46,47]. The velvet gene, *veA* is a global regulatory gene for aflatoxin biosynthesis and conidial and sclerotial morphogenesis [35,48,49].

The objective of the current study was to elucidate the molecular biocontrol mechanisms of *W. anomalus* WRL-076 antagonistic against S- and L-morphotypes of *A. flavus*. Transcription regulation of genes involved in AF biosynthesis, conidiation, and sclerotial production were analyzed. The consequences of these gene expression levels on aflatoxin concentrations in cultural medium, fungal biomass, and number of conidia were determined.

## 2. Results and Discussion

### 2.1. Down Regulation of Expression of AF Biosynthetic Cluster Genes by W. anomalus

**Figure 1:**
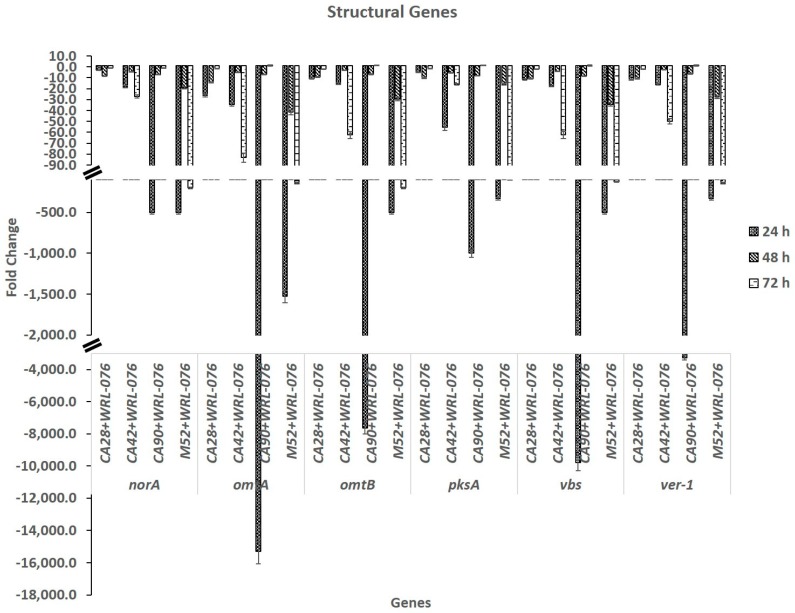
Real time relative quantitative reverse transcription of structural genes by comparing *A. flavus* co-cultured with WRL-076 to *A. flavus* control in fold of changes (y-axis) as the relative expression of *norA*, *omtA*, *omtB, pksA, vbs*, and *ver-1* (x-axis) at 24 h, 48 h, and 72 h.

Eight genes in the AF biosynthesis pathway selected for this study were summarized in Table 1. The expression levels of *aflR* and *aflJ* were decreased a few folds over a time span of 24 h, 48 h, and 72 h (Table 2). Expression of other clustering structural genes such as *pksA*, *norA*, and *omtB* were repressed, ranging from 10 to more than 10,000 fold (Figure 1). The decrease in expression was strain and time dependent. The L-morphotype strains CA90 and M52 showed the most repression in samples collected at 24 h. However, the repression of gene transcription was leveled off at 48 h and 72 h for CA90, but for M52, the repression was observed at 48 h and continued at 72 h. The S-morphotype strains CA28 and CA42 showed a moderate repression on *pksA*, *norA*, *omtA*, *omtB*, *vbs*, and *ver-1* in the range under 100 fold compared to the L-morphotype strains. Apparently, the entire gene cluster of aflatoxin biosynthesis was affected by *W. anomalus*. The global regulatory gene, *veA* was repressed a few fold. The repression level was also strain and time dependent.

### 2.2. Effect of *W.* anomalus on Transcription of Genes of Conidiation and Sclerotial Formation

**Figure 2:**
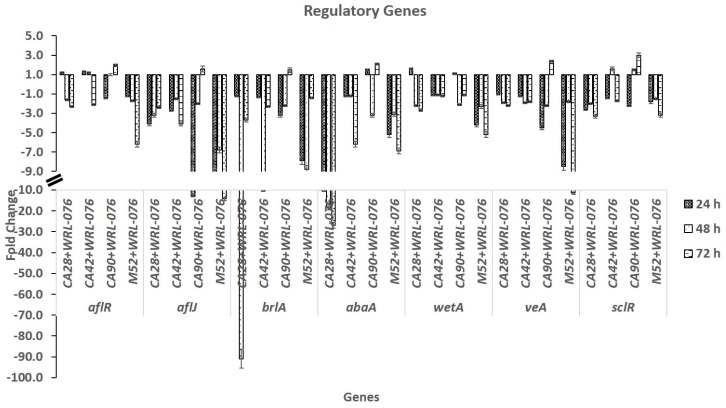
Real time relative quantitative reverse transcription of aflatoxin biosynthesis, conidiation, and sclerotial formation by comparing *A. flavus* co-cultured with WRL-076 to *A. flavus* control in fold of changes (y-axis) as the relative expression of *aflR*, *aflJ*, *brlA*, *abaA*, *wetA*, *veA*, and *sclR* (x-axis) at 24 h, 48 h, and 72 h.

AF biosynthesis and *Aspergillus* development are closely associated processes [38]. We examined the expression levels of *brlA, abaA*, and *wetA* in four *A. flavus* S- and L-strains. The expression levels of three central regulatory genes of conidiation were decreased in co-cultures of *A. flavus* and yeast compared to those from *A. flavus* cultures without yeast. The expression of *brlA* level was several folds lower for the L-morphotype (CA90, M52) at 24 h and 48 h than the S-morphotype, CA28 and CA42. Both had the greatest decrease at 48 h. The *wetA* expression levels had the lowest decreases in comparison to *brlA* and *abaA*. Repression of these three genes in CA28, CA42, CA90, and M52 were variable depending on the strains. The four *A. flavus* strains showed a peak decrease either at 24 h, 48 h, or 72 h (see Figure 2).

*SclR* is a transcription factor for sclerotial formation. The *veA* gene positively regulates the production of aflatoxin and conidial and sclerotial formation. Transcriptional levels of the *veA* gene were repressed in both S and L strains of *A. flavus* (see Figure 2).

### 2.3. Inhibition of Aflatoxin Production in Yeast and *A. flavus* Dual Cultures

We examined the influence of *W. anomalus* on AFB_1_ production on the four toxigenic *A. flavus* strains including two L- and two S-morphotypes [50]. AFB_1_ concentrations of these strains grown in potato dextrose broth (PDB) ranged from 2.6, 3.5, 8.5, to 9.0 µg/culture for CA90, M52, CA42, and CA28, respectively. The two S-morphotypes, CA28 and CA42 produced a higher amount of AFB_1_ than the two L-morphotypes, CA90 and M52. No aflatoxin was detected when these strains were grown in the presence of *W. anomalus* (Table S1). The aflatoxin produced from dual cultures of toxigenic *A. flavus* and *W. anomalus* was significantly lower than that from the toxigenic *A. flavus* control at a *p*-value < 0.05 by ANOVA test. The results demonstrated the yeast biocontrol agent *W. anomalus* WRL-076 is effective in inhibiting aflatoxin biosynthesis.

The decrease of aflatoxin B_1_ concentrations produced by *A. flavus* strains was due to the down regulation of the expression of the entire clustered aflatoxin biosynthetic genes. 

### 2.4. Reduction of Fungal Biomass and Number of Conidial Formation

The fungal mass of CA28, CA42, CA90, and M52 was reduced when co-cultured with *W. anomalus*. The percentage of reduction was 62, 60, 56, and 80%, respectively (Table S2). The percentage of reduction of fungal biomass samples of dual cultures of *A. flavus* with *W. anomalus* was significantly lower than toxigenic *A. flavus* alone at *p*-value < 0.05 by ANOVA Duncan’s multiple range test.

Both CA28 and CA42 strains produced very small numbers of conidia due to their S-morphotype. The numbers of conidia formed in CA28, CA42, CA90, and M52 were 2.1 × 10^4^/mL, 7.1 × 10^5^/mL, 1.5 × 10^7^/mL, and 1.1 × 10^7^/mL, respectively. *W. anomalus* WRL-076 inhibited the spore production in fungal ball biomass; no spore was detected in CA28+WRL-076 and CA42+WRL-076.

The fungal conidia formed on fungal balls after two weeks of dual cultures of *A. flavus* with *W. anomalus* were significantly lower than the *A. flavus* control at a *p*-value < 0.05 by ANOVA test for CA42 and CA42+WRL-076, CA90 and CA90+WRL-076, and M52 and M52+WRL-076. However, the difference between CA28 and CA28+WRL-076 was not significant.

**Figure 3:**
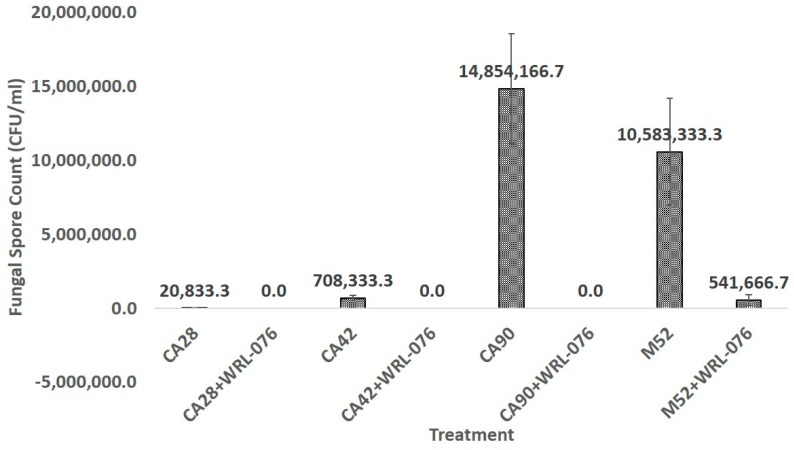
Hemocytometer counts of fungal conidia formed on fungal balls after 2 weeks of incubation at 28 °C. Conidial reduction was calculated in percentage.

*Aspergillus flavus* primarily reproduces by forming asexual spores called conidia, whose formation and maturation are governed by the central genetic regulatory circuit consisting of *BrlA*, *AbaA*, and *WetA*. Genes encoding the regulators were repressed when co-cultured with *W. anomalus*, resulting in a significant reduction of *A. flavus* conidial production (Figure 3, Table S3).

The *veA* gene positively regulates the production of aflatoxin, and conidia and sclerotial formation. Both *veA* and *sclR* were down regulated by *W. anomalus* and their transcriptional levels decreased several folds in both S- and L-strains. We did not detect any sclerotia in CA28 and CA42 (S-strains) and CA90 and M52 (L-strains).

## 3. Conclusions

Transcription of AF biosynthetic genes and conidial regulatory genes in *A. flavus* were both down regulated. Consistent with the decreased gene expression levels, the aflatoxin concentrations in cultural medium were greatly reduced to non-detectable levels. Fungal biomass and the number of conidia were significantly reduced by 60% and more than 95%, respectively. However, the biocontrol yeast cells from fungal ball of dual cultures grew and reached 1 to 2 × 10^8^ CFU /mL (Table S2 and Table S3). The data demonstrate that *W. anomalus* is a robust biocontrol agent.

The Food and Agriculture Organization (FAO) of the United Nations estimates that 25% of the world’s food crops are affected by mycotoxins. Contamination by mycotoxins such as aflatoxin in tree nuts, peanut, corn, and cottonseed is a serious food safety hazard to both humans and animals. The results of this study demonstrate that *W. anomalus* is a promising biocontrol agent to reduce aflatoxin, conidia, and sclerotia of *A. flavus* in agricultural production of crops.

## 4. Material and Methods

### 4.1. Microbial Strains and Media

*W. anomalus* WRL-076 and *A. flavus* strains, CA28, CA42, CA90, and M52, were maintained on potato dextrose agar (PDA, Becton Dickinson & Co., Sparks, MD) at 4 °C. The CA28 and CA42 strains produced small sclerotia (S-morphotype) and CA90 and M52 produced large sclerotia (L-morphotype) as classified by Cotty [50]. Suspensions of yeast and fungal spores were prepared in 0.05% Tween 80 solution and counted using a hemocytometer. Potato dextrose broth (PDB) was the medium used to grow yeast and fungus for investigating the biocontrol antagonistic activities.

### 4.2. Experimental Design

*A. flavus* spores were inoculated into 25 mL of PDB (to a final concentration of 10^5^/mL) and grown at 28 °C in triplicates on a rotary shaker at 150 rpm. For dual culture, yeast (*W. anomalus*) cells and fungal spores in a ratio of 1:1 were used. Fungal hyphae were collected at 24 h, 48 h, and 72 h after inoculation. Yeast cells from dual cultures were separated from the fungal hyphae by filtering through the Cellector tissue sieve with 38.1 µm pore size (VWR Scientific, Brisbane, CA, USA) [25]. The hyphae on the sieve was rinsed with DEPC (0.1% diethylpyrocarbonate) water and transferred to several layers of filter paper with suction, dried, and stored at −80 °C. The processed fungal hyphae were used for total RNA extraction.

Total fungal RNA isolation was carried out by using RNeasy^®^ Plant Mini Kit (Qiagen, Valencia, CA, USA). The RNA samples were treated with Ambion^®^ TURBO DNA-free™ DNase (Ambion, Austin, TX, USA). The purity and concentration of fungal RNA were examined by measuring the absorbance of samples at 260 nm and 280 nm using an ND-1000 Spectrophotometer (NanoDrop Technologies, Wilmington, DE, USA). Samples were stored in a −80 °C freezer. GeneAmp^®^ RNA PCR Core Kit (Applied Biosystems) was used for reverse transcription to obtain cDNA according to the manufacturer’s procedure. For negative control, the same reactions were performed in the absence of the enzyme.

### 4.3. Real Time RT-PCR Analysis of AF Biosynthetic Genes and Conidia Regulatory Genes

Primers were designed with ABI Primer Express 3.0 software (Applied Biosystems, Foster City, CA, USA). Primers for RT-PCR are listed in Table 3 [26,51]. Quantitative PCR reactions were carried out in an ABI 7300 Real Time PCR System. SYBR^®^ Green PCR Master Mix (Applied Biosystems, Foster City, CA USA), which increases fluorescence upon binding to double-stranded DNA product, was used as the amplification detector. Triplicates of each reaction were performed. The final primer concentration was 500 nM in the 25 μL reaction mixture. Input cDNA quantities in the reaction mixture were within the recommended 150 ng. The PCR cycles were programmed as follows—2 min at 50 °C for AmpErase^®^ UNG Activation, 10 min at 95 °C for AmpliTaq Gold ^®^ DNA polymerase activation, followed by 40 cycles of 15 s at 95 °C and 1 min at 60 °C for both primer annealing and product extension. Melting curve analysis was performed using Dissociation Curves software (Applied Biosystems) to ensure only a single product was amplified. Amplification of *A. flavus* 18S ribosomal RNA was used as the endogenous control (reference gene) due to its relatively stable expression level. Plates and quantification assay documents were created in SDS^®^ Software 1.3.1 (Applied Biosystems). The relative quantification of gene expression changes was computed by using the 2^ΔΔCt^ method [52,53,54].

### 4.4. High Performance Liquid Chromatography (HPLC) Analysis of AFB_1_

AFB_1_ was extracted from a 2 mL liquid fungal and yeast co-culture by adding 1 mL of acetonitrile into the conical tube, vortex for 10 min, and 0.5 mL of the supernatant was filtered through SINGLE StEP^TM^ eXtreme/FV 0.45 mm Nylon (Thomson Instrument Company; Oceanside, CA). Filtered samples were analyzed by high performance liquid chromatography (HPLC) on an Agilent model 1260 Infinity ChemStation (Agilent, Palo Alto, California, USA). HPLC was performed on a Supelcosil LC-18 reversed-phase column (150 mm × 4.6 mm i.d., 5 μm particle size) at a flow rate of 1 mL/min.

The mobile phase was methanol/acetonitrile/H_2_O (20:20:60). Aflatoxins were quantified by a fluorescent detector with excitation at 365 nm and emission at 455 nm and quantified by peak areas relative to a standard curve of authentic AFB_1_ [55]. Aflatoxin standards were purchased from Sigma-Aldrich (St. Louise, MO, USA).

### 4.5. Determination of Fungal Biomass and Conidia Numbers

*A. flavus* hyphae grown in PDB with shaking in triplicate flasks formed tiny fungal balls and increased in size over the time of incubation. Dual cultures of *A. flavus* and *W. anomalus* also formed fungal balls. After incubating the cultures for 72 h, fungal balls from each flask with and without *W. anomalus* were collected on a meshed screen rinsed with sterile water and transferred to an empty Petri Dish to induce conidiation. The harvested fungal balls were weighed and then incubated at 28 °C for two weeks [25]. Conidia (spore) were then extracted in 5 mL of 0.05% Tween 80 solution, and conidia and yeast cells were counted using a hemocytometer.

### 4.6. Statistical Analysis

Statistical analyses were performed with SAS Enterprise Guide (version 6.1, SAS Institute Inc., Cary, NC, USA). ANOVA (one-way analysis of variance) by Duncan’s multiple range test at a 95% confidence level (*p*-value < 0.05) was performed on all the samples.

## Figures and Tables

**Table 1:** Functions of genes used in this study.

Gene	Enzyme/Product	Functions
*aflR*	transcription activator AflR	pathway-specific regulator
*aflJ*	transcription enhancer	pathway co-regulator
*norA*	norsolorinic acid reductase	NOR → AVN
*omtA*	O-methyltransferase A	ST → OMST
*omtB*	O-methyltransferase B	DHDMST → DHST
*pksA*	polyketide synthase (PKS)	acetate → polyketide
*vbs*	versicolorin B synthase	VAL → VERB
*ver-1*	dehydrogenase/ketoreductase	VERA → DMST
*brlA*	C_2_H_2_ zinc finger transcription factor	control of conidiophore development
*abaA*	conidial formation (abacus)	conidiation regulator activated by BrlA
*wetA*	conidial formation (wet-white conidia)	conidiation regulator activated by AbaA
*veA*	velvet nuclear complex component	global regulator
*sclR*	sclerotial formation	sclerotium regulator

Abbreviations—NOR, norsolorinic acid; AVN, averantin; VHA, versicon hemiacetal acetate; VAL, versiconal; VERB, versicolorin B; DHDMST, dihydrodemethylsterigmatocystin; DHST, dihydrosterigmatocystin; ST, sterigmatocystin; OMST, O-methylsterigmatocystin; DHOMST, dihydro-O-methylsterigmatocystin (Yu et al., 2004; Nieman et al., 2015).

**Table 2:** Real time RT-PCR assays of aflatoxin biosynthetic pathway and conidiation/sclerotial genes at 24 h, 48 h, and 72 h in potato dextrose broth (PDB).

Gene	CA28+WRL-076			CA42+WRL-076			CA90+WRL-076			M52+WRL-076		
	24 h	48 h	72 h	24 h	48 h	72 h	24 h	48 h	72 h	24 h	48 h	72 h
*aflR*	1.2 ± 0.1	−1.6 ± 0.1	−2.3 ± 0.1	1.3 ± 0.1	1.2 ± 0.1	−2.1 ± 0.1	−1.4 ± 0.1	1.0 ± 0.1	2.0 ± 0.1	−1.2 ± 0.1	−1.7 ± 0.1	−6.2 ± 0.3
*aflJ*	−4.1 ± 0.2	−3.2 ± 0.2	−2.4 ± 0.1	−2.7 ± 0.1	−1.5 ± 0.1	−4.1 ± 0.2	−12.7 ± 0.6	−2.0 ± 0.1	1.6 ± 0.3	−9.3 ± 0.5	−6.8 ± 0.3	−14.3 ± 0.7
*norA*	−2.8 ± 0.1	−8.5 ± 0.4	−1.1 ± 0.1	−18.2 ± 0.9	−4.5 ± 0.2	−27.0 ± 1.4	−500.0 ± 25.0	−7.1 ± 0.4	−1.0 ± 0.1	−500.0 ± 25.0	−19.2 ± 1.0	−200.0 ± 10.0
*omtA*	−26.3 ± 1.3	−14.3 ± 0.7	−1.7 ± 0.1	−34.5 ± 1.7	−5.2 ± 0.3	−83.3 ± 4.2	−15,290.5 ± 764.5	−6.9 ± 0.3	1.2 ± 0.1	−1,526.7 ± 76.3	−41.7 ± 2.1	−142.9 ± 7.1
*omtB*	−10.8 ± 0.5	−9.3 ± 0.5	−1.8 ± 0.1	−15.4 ± 0.8	−3.1 ± 0.2	−62.5 ± 3.1	−7,633.6 ± 381.7	−6.8 ± 0.3	1.3 ± 0.1	−500.0 ± 24.9	−29.4 ± 1.5	−200.0 ± 9.8
*pksA*	−5.2 ± 0.3	−10.4 ± 0.5	−1.6 ± 0.1	−55.6 ± 2.8	−5.5 ± 0.3	−16.1 ± 0.8	−1,000.0 ± 50.0	−8.1 ± 0.4	1.4 ± 0.1	−333.3 ± 16.7	−15.9 ± 0.8	−100.0 ± 5.0
*vbs*	−11.5 ± 0.6	−11.0 ± 0.5	−1.8 ± 0.1	−17.5 ± 0.9	−3.9 ± 0.2	−62.5 ± 3.1	−9,803.9 ± 490.2	−8.5 ± 0.4	1.2 ± 0.1	−500.0 ± 24.8	−34.5 ± 1.7	−125.0 ± 6.3
*ver−1*	−11.9 ± 0.6	−10.9 ± 0.5	−2.1 ± 0.1	−16.1 ± 0.8	−2.5 ± 0.1	−50.0 ± 2.5	−3,257.3 ± 162.9	−6.3 ± 0.3	1.2 ± 0.1	−333.3 ± 16.4	−27.8 ± 1.4	−142.9 ± 7.3
*brlA*	−1.2 ± 0.1	−90.9 ± 4.5	−3.7 ± 0.2	−1.3 ± 0.1	−10.3 ± 0.5	−2.3 ± 0.1	−3.2 ± 0.2	−2.2 ± 0.1	1.5 ± 0.2	−7.9 ± 0.4	−8.8 ± 0.4	−1.4 ± 0.1
*abaA*	−10.2 ± 0.5	−18.5 ± 0.9	−27.0 ± 1.4	−1.2 ± 0.1	−1.2 ± 0.1	−6.2 ± 0.3	1.5 ± 0.1	−3.2 ± 0.2	2.1 ± 0.1	−5.2 ± 0.3	−3.1 ± 0.2	−6.9 ± 0.3
*wetA*	1.6 ± 0.1	−2.2 ± 0.1	−2.7 ± 0.1	−1.1 ± 0.1	−1.1 ± 0.1	−1.2 ± 0.1	1.1 ± 0.1	−2.1 ± 0.1	−1.1 ± 0.1	−4.2 ± 0.2	−2.3 ± 0.2	−5.2 ± 0.3
*veA*	−1.0 ± 0.1	−1.9 ± 0.1	−2.2 ± 0.1	−1.2 ± 0.1	−1.9 ± 0.1	−1.8 ± 0.1	−4.5 ± 0.2	−2.2 ± 0.1	2.4 ± 0.1	−8.5 ± 0.4	−1.8 ± 0.1	−11.5 ± 0.6
*sclR*	−2.6 ± 0.1	−2.0 ± 0.1	−3.3 ± 0.2	−1.4 ± 0.1	1.6 ± 0.2	−1.7 ± 0.1	−2.2 ± 0.1	1.5 ± 0.1	3.0 ± 0.2	−1.8 ± 0.2	−1.5 ± 0.1	−3.2 ± 0.2

**Table 3:** PCR primers used in this study.

Gene	Forward Primer	Reverse Primer
*18S*	TTCCTAGCGAGCCCAACCT	GCCCGCCGAAGCAACT
*aflR*	GCCGCGCCCGAAA	GCACTTTTGAGCTGGCACAA
*aflJ*	CCGAAGATTCCGCTTGGA	TGAAGACATGCAGCAAAAGGA
*norA*	TCTAGCGCCGGTGTTCGT	CATTGCCGAAGCTCATCGTT
*omtA*	CGGGTTTCGCAAAAGCAT	GCAGGCAGGTCCTGTACGA
*omtB*	TGCTGTGGCATCCATTCAAA	GGACTGCGTCTTCCAAAAGG
*pksA*	TCACAAGCGATGCACAGTTG	AACTGACGAATGTGGGTCTTGTACT
*vbs*	GAGTCTACCGCCGCCGATA	GAAAAGGTCGGCCAGTCATC
*ver-1*	GGTCCCCAAGCATGCTGTA	GCAGCGAACAAAGGTGTCAAT
*brlA*	TCAAGACGCACAGCCCTACA	GACGCGGTGCCGATAGAG
*abaA*	GAGTGGCAGACCGAATGTATGTTG	TAGTGGTAGGCATTGGGTGAGTTG
*wetA*	CCACAGCAGCCGATCCA	CCCCTTGCAGGATGTCATG
*veA*	TGGACCGCCCATCTCAAG	ATGCCGCACGGAAAGATC
*sclR*	TGCCGCACACAACATCATT	TTCTCCAAGGCCACGAACTT

## Supplementary Materials

The following are available online at http://www.mdpi.com/2072-6651/11/2/81/s1. Table S1. High performance liquid chromatography (HPLC) data and aflatoxin calculation. Table S2. Fungal biomass evaluation of *A. flavus* and *A. flavus* co-cultured with *W. anomalus*. Table S3. Hemocytometer counts of yeast and fungal conidia formed on fungal balls after 2 weeks of incubation.

## References

[1] Henry S.H., Bosch F.X., Bowers J.C. (2002). Aflatoxin, hepatitis and worldwide liver cancer risks. Adv. Exp. Med. Biol..

[2] Hedayati M.T., Pasqualotto A.C., Warn P.A., Bowyer P., Denning D.W. (2007). *Aspergillus flavus*: Human pathogen, allergen and mycotoxin producer. Microbiology.

[3] Molyneux R.J., Mahoney N., Kim J.H., Campbell B.C. (2007). Mycotoxins in edible tree nuts. Int. J. Food Microbiol..

[4] Trucksess M.W., Scott P.M. (2008). Mycotoxins in botanicals and dried fruits: A review. Food Addit. Contam. Part A Chem. Anal. Control. Expo. Risk Assess..

[5] Amaike S., Keller N.P. (2011). *Aspergillus* *flavus*. Annu. Rev. Phytopathol..

[6] Roze L.V., Hong S.-Y., Linz J.E. (2013). Aflatoxin Biosynthesis: Current Frontiers. Annu. Rev. Food Sci. Technol..

[7] Hua S.S.T., Chang P.K., Palumbo J.D., Witczak A., Sikorski Z.E. (2017). Mycotoxins. Toxins and Other Harmful Compounds in Foods.

[8] Van Egmond H.P., Schothorst R.C., Jonker M.A. (2007). Regulations relating to mycotoxins in food: Perspectives in a global and European context. Anal. Bioanal. Chem..

[9] Food and Agriculture Organization (FAO) (2009). Declaration of the World Summit on Food Security.

[10] Commission of the European Community (1998). Commission Directive 98/53/EC of July 1998 laying down the sampling methods and the methods of analysis for the official control of the levels of certain contaminants in food stuffs. Off. Eur. Commun. Legis..

[11] Kurtzman C.P. (2011). Recognition of Yeast Species from Gene Sequence Comparisons. Open Appl. Inform. J..

[12] Petersson S., Schnurer J. (1995). Biocontrol of mold growth in high-moisture wheat stored under airtight conditions by *Pichia anomala*, *Pichia guilliermondii*, and *Saccharomyces cerevisiae*. Appl. Environ. Microbiol..

[13] Petersson S., Schnurer J. (1998). *Pichia anomala* as a biocontrol agent of *Penicillium roqueforti* in high-moisture wheat, rye, barley, and oats stored under airtight conditions. Can. J. Microbiol..

[14] Schnürer J., Jonsson A. (2011). *Pichia anomala* J121: A 30-year overnight near success biopreservation story. Antonie Van Leeuwenhoek Int. J. Gen. Mol. Microbiol..

[15] Jijakli M.H., Lepoivre P. (1998). Characterization of an *Exo-beta-1,3-Glucanase* Produced by *Pichia anomala* Strain K, Antagonist of *Botrytis cinerea* on Apples. Phytopathology.

[16] Haïssam J.M. (2011). *Pichia anomala* in biocontrol for apples: 20 years of fundamental research and practical applications. Antonie Van Leeuwenhoek Int. J. Gen. Mol. Microbiol..

[17] Petersson S., Hansen M.W., Axberg K., Hult K., Schnürer J. (1998). Ochratoxin a accumulation in cultures of *Penicillium verrucosum* with the antagonistic yeast *Pichia anomala* and *Saccharomyces cerevisiae*. Mycol. Res..

[18] Hua S.S.T., Baker J.L., Flores-Espiritu M. (1999). Interactions of saprophytic yeasts with a nor mutant of *Aspergillus flavus*. Appl. Environ. Microbiol..

[19] Hua S.S.T., Battle I., Hormaza I., Espiau M.T. (2002). Potential use of saprophytic yeast to reduce populations of *Aspergillus flavus* in almond and pistachio orchards. Proceedings of the Third International Symposium of Pistachio and Almond.

[20] Hua S.S.T. (2004). Application of a yeast, *Pichia anomala* strain WRL-076 to control *Aspergillus flavus* for reducing aflatoxin in pistachio and almond. IOBC Bulletin.

[21] Hua S.S.T., Parfitt D.E., Holtz B.A. Evaluation of a biopesticide, *Pichia anomala* WRL-076 to control *Aspergillus flavus* in a commercial orchard. Proceedings of the California Conference of Biological Control V.

[22] Hua S.S.T., Mendez-Vilas A. (2013). Biocontrol of Aspergillus flavus by Pichia anomala. Microbial Pathogens and Strategies for Combating Them: Science, Technology and Education.

[23] Isakeit T., Bétran F.J., Odvody G., Hua S.S.T. (2005). Efficacy of *Pichia anomala* WLR-076 to control aflatoxin on corn in Texas. https://www.ars.usda.gov/research/publications/publication/?seqNo115=223081.

[24] Hua S.S.T., Hernlem B.J., Yokoyama W., Sarreal S.B.L. (2015). Intracellular trehalose and sorbitol synergistically promoting cell viability of a biocontrol yeast, *Pichia anomala*, for aflatoxin reduction. World J. Microbiol. Biotechnol..

[25] Hua S.S.T., Brandl M., Hernlem B., Eng J.G., Sarreal S.B.L. (2011). Fluorescent viability stains to probe the metabolic status of aflatoxigenic fungus in dual culture of *Aspergillus flavus* and *Pichia anomala*. Mycopathologia.

[26] Hua S.S.T., Beck J.J., Sarreal S.B.L., Gee W. (2014). The major volatile compound 2-phenylethanol from the biocontrol yeast, *Pichia anomala*, inhibits growth and expression of aflatoxin biosynthetic genes of *Aspergillus flavus*. Mycotoxin Res..

[27] Chang P.K., Hua S.S.T., Sarreal S.B.L., Li R.W. (2015). Suppression of aflatoxin biosynthesis in *Aspergillus flavus* by 2-phenylethanol is associated with stimulated growth and decreased degradation of branched-chain amino acids. Toxins.

[28] Bayman P., Cotty P.J. (1993). Genetic diversity in *Aspergillus flavus*: Association with aflatoxin production and morphology. Can. J. Bot..

[29] Ehrlich K.C., Montalbano B.G., Cotty P.J. (2005). Divergent regulation of aflatoxin production at acidic pH by two *Aspergillus* strains. Mycopathologia.

[30] Ohkura M., Cotty P.J., Orbach M.J. (2018). Comparative genomics of *Aspergillus flavus* S and L morphotypes yield Insights into niche adaptation. G3 (Bethesda).

[31] Yu J., Chang P.K., Ehrlich K.C., Cary J.W., Bhatnagar D., Cleveland T.E., Payne G.A., Linz J.E., Woloshuk C.P., Bennett J.W. (2004). Clustered Pathway Genes in Aflatoxin Biosynthesis. Appl. Environ. Microbiol..

[32] Ehrlich K.C., Yu J., Cotty P.J. (2005). Aflatoxin biosynthesis gene clusters and flanking regions. J. Appl. Microbiol..

[33] Yu J. (2012). Current understanding on aflatoxin biosynthesis and future perspective in reducing aflatoxin contamination. Toxins (Basel).

[34] Ehrlich K.C., Montalbano B.G., Cotty P.J. (2003). Sequence comparison of *aflR* from different *Aspergillus* species provides evidence for variability in regulation of aflatoxin production. Fungal Genet. Biol..

[35] Calvo A.M., Bok J., Brooks W., Keller N.P. (2004). *veA* is required for toxin and sclerotial production in *Aspergillus parasiticus*. Appl. Environ. Microbiol..

[36] Calvo A.M. (2008). The VeA regulatory system and its role in morphological and chemical development in fungi. Fungal Genet. Biol..

[37] Georgianna D.R., Payne G.A. (2009). Genetic regulation of aflatoxin biosynthesis: From gene to genome. Fungal Genet. Biol..

[38] Amare M.G., Keller N.P. (2014). Molecular mechanisms of *Aspergillus flavus* secondary metabolism and development. Fungal Genet. Biol..

[39] Adams T.H., Boylan M.T., Timberlake W.E. (1988). *brlA* is necessary and sufficient to direct conidiophore development in *Aspergillus nidulans*. Cell.

[40] Adams T.H., Wieser J.K., Yu J.H. (1998). Asexual sporulation in *Aspergillus nidulans*. Microbiol. Mol. Biol. Rev..

[41] Andrianopoulos A., Timberlake W.E. (1994). The *Aspergillus nidulans abaA* gene encodes a transcriptional activator that acts as a genetic switch to control development. Mol. Cell. Biol..

[42] Marshall M.A., Timberlake W.E. (1991). *Aspergillus nidulans wetA* activates spore-specific gene expression. Mol. Cell. Biol..

[43] Etxebeste O., Garzia A., Espeso E.A., Ugalde U. (2010). *Aspergillus nidulans* asexual development: Making the most of cellular modules. Trends Microbiol..

[44] Wu M.Y., Mead M.E., Kim S.C., Rokas A., Yu J.H. (2017). WetA bridges cellular and chemical development in *Aspergillus flavus*. PLoS ONE.

[45] Jin F.J., Takahashi T., Matsushima K.I., Hara S., Shinohara Y., Maruyama J.I., Kitamoto K., Koyama Y. (2011). SclR, a basic helix-loop-helix transcription factor, regulates hyphal morphology and promotes sclerotial formation in *Aspergillus oryzae*. Eukaryot. Cell.

[46] Wicklow D.T. (1987). Survival of *Aspergillus flavus* sclerotia in soil. Trans. Br. Mycol. Soc..

[47] Wicklow D.T., Wilson D.M., Nelsen T.C. (1993). Survival of *Aspergillus-Flavus* Sclerotia and Conidia Buried in Soil in Illinois or Georgia. Phytopathology.

[48] Cary J.W., OBrian G.R., Nielsen D.M., Nierman W., Harris-Coward P., Yu J., Bhatnagar D., Cleveland T.E., Payne G.A., Calvo A.M. (2007). Elucidation of *veA*-dependent genes associated with aflatoxin and sclerotial production in *Aspergillus flavus* by functional genomics. Appl. Microbiol. Biotechnol..

[49] Duran R.M., Cary J.W., Calvo A.M. (2007). Production of cyclopiazonic acid, aflatrem, and aflatoxin by *Aspergillus flavus* is regulated by *veA*, a gene necessary for sclerotial formation. Appl. Microbiol. Biotechnol..

[50] Cotty P.J. (1997). Aflatoxin-producing potential of communities of *Aspergillus section Flavi* from cotton producing areas in the United States. Mycol. Res..

[51] Chang P.K., Scharfenstein L.L., Mack B., Ehrlich K.C. (2012). Deletion of the *Aspergillus flavus* orthologue of *A. Nidulans fluG* reduces conidiation and promotes production of sclerotia but does not abolish aflatoxin biosynthesis. Appl. Environ. Microbiol..

[52] Livak K.J., Schmittgen T.D. (2001). Analysis of relative gene expression data using real-time quantitative PCR and the 2-ΔΔCT method. Methods.

[53] Pfaffl M.W. (2001). A new mathematical model for relative quantification in real-time RT-PCR. Nucleic Acids Res..

[54] Pfaffl M. (2004). Quantification strategies in real-time PCR. A–Z of Quantitative PCR.

[55] Hua S.S.T., Palumbo J.D., Parfitt D., Sarreal S.L., O’Keeffe T. (2018). Development of a droplet digital PCR assay for population analysis of aflatoxigenic and atoxigenic *Aspergillus flavus* mixtures in soil. Mycotoxin Res..

